# UV bullseye contrast of *Hemerocallis* flowers attracts hawkmoths but not swallowtail butterflies

**DOI:** 10.1002/ece3.4604

**Published:** 2018-12-18

**Authors:** Shun K. Hirota, Nozomu Miki, Akiko A. Yasumoto, Tetsukazu Yahara

**Affiliations:** ^1^ Field Science Center, Graduate School of Agricultural Science Tohoku University Osaki Japan; ^2^ Graduate School of Systems Life Sciences Kyushu University Fukuoka Japan; ^3^ Department of Evolutionary Biology and Environmental Studies University of Zurich Zurich Switzerland; ^4^ Department of Biology, Faculty of Science Kyushu University Fukuoka Japan

**Keywords:** butterflies, color contrast, floral evolution, flower color, hawkmoths, pollinator behavior, UV bullseye floral pattern

## Abstract

The color and patterns of animal‐pollinated flowers are known to have effects on pollinator attraction. In this study, the relative importance of flower color and color contrast patterns on pollinator attraction was examined in two pollinator groups, swallowtail butterflies and hawkmoths using two *Hemerocallis* species; butterfly‐pollinated *H. fulva* and hawkmoth‐pollinated *H. citrina,* having reddish and yellowish flowers in human vision, respectively. Flowers of both species have UV bullseye patterns, composed of UV‐absorbing centers and UV‐reflecting peripheries, known to function as a typical nectar guide, but UV reflectance was significantly more intense in the peripheries of *H. citrina* flowers than in those of *H. fulva *flowers. Comparison based on the visual systems of butterflies and hawkmoths showed that the color contrast of the bullseye pattern in *H. citrina* was more intense than that in *H. fulva*. To evaluate the relative importance of flower color and the color contrast of bullseye pattern on pollinator attraction, we performed a series of observations using experimental arrays consisting of *Hemerocallis* species and their hybrids. As a result, swallowtail butterflies and crepuscular/nocturnal hawkmoths showed contrasting preferences for flower color and patterns: butterflies preferred *H. fulva*‐like colored flower whereas the preference of hawkmoths was affected by the color contrast of the bullseye pattern rather than flower color. Both crepuscular and nocturnal hawkmoths consistently preferred flowers with stronger contrast of the UV bullseye pattern, whereas the preference of hawkmoths for flower color was incoherent. Our finding suggests that hawkmoths can use UV‐absorbing/reflecting bullseye patterns for foraging under light‐limited environments and that the intensified bullseye contrast of *H. citrina* evolved as an adaptation to hawkmoths. Our results also showed the difference of visual systems between pollinators, which may have promoted floral divergence.

## 1 INTRODUCTION

Since Darwin (1862) described the hawkmoth‐pollinated orchid with a long floral spur as a notable consequence of natural selection, hawkmoth‐pollinated flowers have been interesting as a model of floral evolution mediated by pollinators. It is now widely known that hawkmoth‐pollinated flowers have a unique set of floral traits including pale colored petals, a sweet floral scent, and long flower tubes or spurs (Grant, 1983, 1985 ; Willmer, 2011), often markedly differentiated from related species pollinated by hummingbirds (Campbell, 2004; Hodges, Fulton, Yang, & Whittall, 2003), swallowtail butterflies (Hirota et al., 2012), bees (Hoballah et al., 2007) or long‐tongued flies (Johnson, 2006). To deepen our understanding of the pollinator‐mediated floral divergence, we need to compare the preferences for each floral trait between hawkmoths and other pollinators.

Regarding flower color, many hawkmoth‐pollinated flowers are uniformly white and lacking UV reflectance (Raguso, Henzel, Buchmann, & Nabhan, 2003; White, Stevenson, Bennett, Cutler, & Haber, 1994). Hawkmoths have a trichromatic color vision (UV, blue, green) and, thus can perceive UV wavelength (Höglund, Hamdorf, & Rosner, 1973; Schwemer & Paulsen, 1973; White, Xu, Munch, Bennett, & Grable, 2003). Experimental studies showed that hawkmoths prefer human white color to other colors (Dell'Olivo & Kuhlemeier, 2013; Dell'Olivo, Hoballah, Gübitz, & Kuhlemeier, 2011; Hoballah et al., 2005) and uniformly UV‐absorbing flowers to uniformly UV‐reflecting flowers (White et al., 1994). In contrast, some hawkmoth‐pollinated flowers are yellow with UV bullseye patterns composed of a UV‐absorbing center and a UV‐reflecting periphery as in *Hemerocallis citrina* (Hirota et al., 2012) and *Oenothera *spp. (Kawano et al., 1995; Moody‐Weis & Heywood, 2001). Previous studies showed that UV bullseye patterns are attractive to diurnal pollinators such as bees and syrphid flies (e.g., Koski & Ashman, 2014) and bees make their first antennal contact preferably at the UV‐absorbing part (Papiorek et al., 2016). Nocturnal hawkmoths can use a bullseye‐like pattern of artificial flowers painted with blue and white (Kelber, 2002). The UV bullseye pattern of hawkmoth‐pollinated flowers may act as an advertisement for hawkmoths, but no previous study has been conducted to test the effect of the UV bullseye pattern on hawkmoths.

The two *Hemerocallis* species, diurnal *H. fulva* and nocturnal *H. citrina,* both have flowers with a UV bullseye pattern. *Hemerocallis fulva*, a butterfly‐pollinated species, has diurnal, reddish or orange‐colored flowers in human vision without perceivable scent and *H. citrina*, a hawkmoth‐pollinated species, has nocturnal flowers with yellow color in human vision and a sweet scent in human olfaction. In an experimental array of *Hemerocallis* (Hirota et al., 2013), hawkmoths showed neither constant preference for human red or yellow color nor any preference for floral scent intensity, corolla orientation, and stem height. Thus, it is likely that hawkmoths use any other floral trait differing between the two species of *Hemerocallis* as a foraging cue for detecting the flowers. Between *H. fulva* and *H. citrina*, not only human visible flower color but also the floral UV reflectance is different: the intensity of UV reflectance in the peripheral part is stronger in *H. citrina* than in *H. fulva* (Hirota et al., 2012, Figures 1 and 2). Furthermore, there is evidence that pollinators use not only overall color but also color contrast as foraging cue (Chittka & Raine, 2006; Schmidt, Schaefer, & Winkler, 2004). This might imply that the color contrast between central and peripheral parts (“bullseye contrast”) acts as an efficient advertisement for hawkmoths.

**Figure 1:**
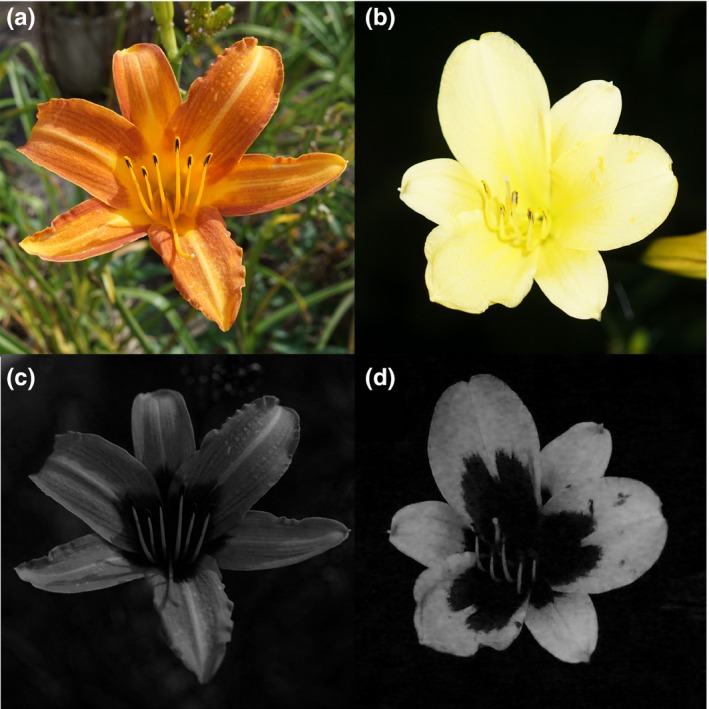
Flowers of *H. fulva* (a,c) and *H. citrina* (b,d). The upper and lower photos represent both flowers under human visible and UV spectrum, respectively. Photographs were taken by a digital camera α6000 (SONY, removed low‐pass filter) with SIGMA 30 mm 2.8 EX DN and NEXCC +U340 filter (HOYA)

**Figure 2:**
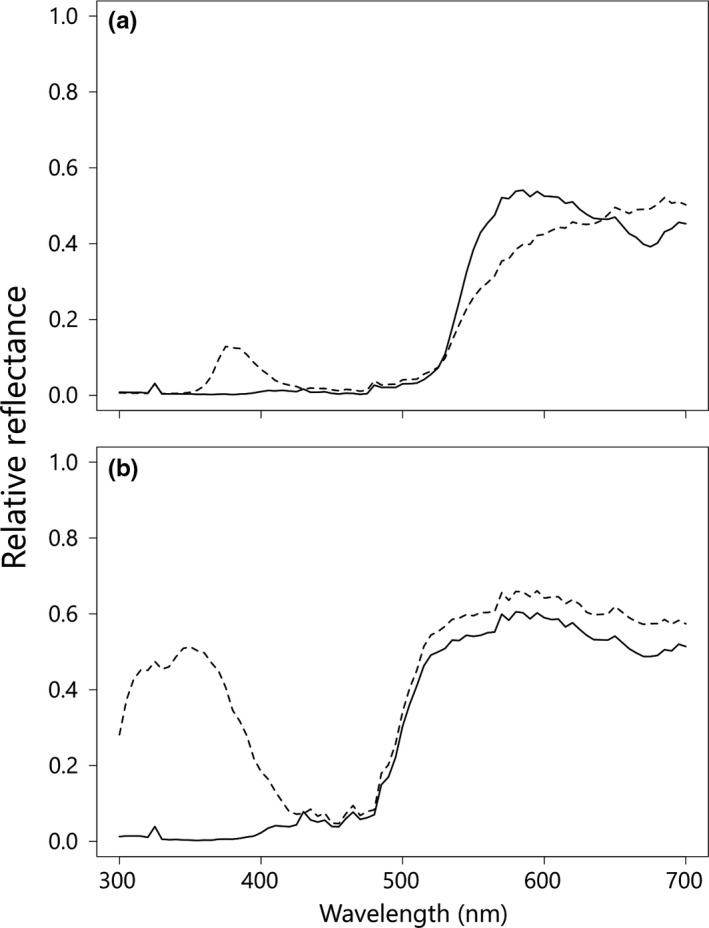
Reflectance spectra in the central part (solid lines) and the peripheral part (dashed lines) of tepals of *H. fulva* (a) and *H. citrina* (b)

As opposed to the trichromatic color vision of hawkmoths, swallowtail butterflies of *Papilio* have a tetrachromatic color vision (UV, blue, green, red), which enables them to discriminate differences in color with higher accuracy (Koshitaka, Kinoshita, Vorobyev, & Arikawa, 2008). In addition, swallowtail butterflies can perceive the color of smaller targets than the trichromatic honeybees (Takeuchi, Arikawa, & Kinoshita, 2006). Thus, flower color may be a more reliable foraging cue for butterflies than for hawkmoths. In laboratory condition, naive individuals of swallowtail butterflies *P. xuthus* preferred more reddish colors when the background was green (Kinoshita, Shimada, & Arikawa, 1999). In the wild, swallowtail butterflies showed a significant preference for human reddish flowers over human yellowish ones while they also preferred weaker scent and taller stems (Hirota et al., 2012, 2013). In contrast, little is known about the preference of butterflies for the floral UV bullseye pattern.

*Hemerocallis fulva* and *H. citrina* provide an extraordinary opportunity to test independent effects of overall flower color and UV bullseye pattern on the attraction of diurnal and nocturnal pollinators. Artificial F2 hybrids show highly variable floral traits, ranging from the floral traits of *H. fulva* to those of *H. citrina* (Hirota et al., 2012, 2013 ; Nitta, Yasumoto, & Yahara, 2010). We carried out a series of trait measurements and field experiments using *H. citrina*, *H. fulva*, and their hybrids, aiming at assessing the relative role of floral traits in attracting swallowtail butterflies and hawkmoths and at testing the hypothesis that hawkmoths use the stronger bullseye contrast as a foraging cue more consistently than other floral traits. First, for parental species and F2 hybrids, spectral properties of central and peripheral flower colors were measured and mapped on a color space defined based on relative excitations of photoreceptors of a swallowtail butterfly and a hawkmoth. Distance in each color space was used as a measure of color contrast. Second, the preferences of butterflies and hawkmoths were assessed using the experimental array consisting of F2 hybrids of *H. fulva* and *H. citrina*. More specifically, we addressed the following questions. (a) How do flower color and color contrast differ between *H. fulva* and *H. citrina* based on butterfly and hawkmoth color vision? (b) Do butterflies and hawkmoths prefer the flowers with the stronger contrast of bullseye pattern? (c) Which of flower color and bullseye pattern is more important as a foraging cue for butterflies and hawkmoths?

## 2 MATERIAL AND METHODS

### 2.1 Study system

*Hemerocallis fulva* L. var. *aurantiaca* (Baker) M. Hotta and *H. citrina *var. *vespertina *(H. Hara) M. Hotta are perennial plants distributed mainly in western Japan, having partly self‐incompatible, hermaphroditic flowers. Corolla of the two species has UV bullseye pattern and the peripheral part of tepals of *H. citrina* reflects UV stronger than that of *H. fulva* (Hirota et al., 2012, Figures 1 and 2). Flower longevity of the two species is approximately half a day; *H. fulva* flowers in the daytime while *H. citrina* flowers at night. The flowering time of the two species overlaps from 16:30 to 21:30 (Nitta et al., 2010). From 16:30 to sunset, both swallowtail butterflies and hawkmoths actively forage on flower nectar of the two species and their hybrids (Hirota et al., 2012). The F1 interspecific hybrids show only weak sterility and can be backcrossed (Yasumoto & Yahara, 2006), and natural hybrid populations with various intermediates have been known in some localities (Hasegawa, Yahara, Yasumoto, & Hotta, 2006). Artificial F2 hybrids show highly variable floral traits across the trait ranges between *H. fulva* and *H. citrina* and various combinations of flower color and scent intensity: for example, human reddish flower with sweet scent or human yellowish flower without scent (Hirota et al., 2012, 2013).

### 2.2 Plant materials

Plants of *H. fulva* and *H. citrina* were collected in Haifuku (Hirado island, Nagasaki Prefecture, Japan) and Tsutsumi about 10 km northeast of Haifuku, respectively (for details, see Yasumoto & Yahara, 2006). To produce F1 hybrids, *H. fulva* plants were hand‐pollinated by pollen of *H. citrina* in 2001 (Yasumoto & Yahara, 2008). To produce F2 hybrids, F1 plants were hand‐pollinated by pollen of full sibling F1 plants in 2003 and 2004 (Nitta et al., 2010). All plants were grown in pots in the nursery of the Department of Biology, Kyushu University (Fukuoka, Japan).

### 2.3 Measurements of flower color and its traits

#### 2.3.1 Mapping floral spectral reflectance into insect color space

The spectral reflectance (300–700 nm) of the central and peripheral parts of individual flowers was measured with a spectrometer (BRC112; B&W Tek Inc., Delaware, USA) relative to a white reflection standard (RS50; StellarNet Inc., Florida, USA) under a deuterium/tungsten light source (BDS130; B&W Tek Inc., Delaware, USA).

Swallowtail butterflies (*Papilio xuthus, Papilio memnon*, and *Papilio helenus*) and crepuscular hawkmoths (*Theretra japonica*, *Theretra oldenlandiae,* and *Theretra silhetensis*) are effective pollinators of *H. fulva* and *H. citrina*, respectively (Hirota et al., 2012, 2013). Because of the tetrachromatic vision of *P. xuthus* (Koshitaka et al., 2008), we employed a tetrachromatic model for swallowtail butterflies. Due to the trichromatic vision of the crepuscular hawkmoth *Deilephila elpenor* (Höglund et al., 1973; Schwemer & Paulsen, 1973), we employed a trichromatic model for hawkmoths. We translated the reflectance spectra into chromatic representations of *P. xuthus* and *D. elpenor* using the following equation (Chittka & Kevan, 2005).

$$P_{i}=\;\frac{\int_{300}^{700}S_{i}\left(\lambda \right)\cdot I_{S}\left(\lambda \right)\cdot D_{65}\left(\lambda \right)\text{d}\lambda }{\int_{300}^{700}S_{i}\left(\lambda \right)\cdot I_{B}\left(\lambda \right)\cdot D_{65}\left(\lambda \right)\text{d}\lambda },$$

where *P_i_* is the relative number of quanta absorbed by the photoreceptor class *i* of *P. xuthus* (UV, blue, green, and red) or *D. elpenor *(UV, blue, green), *S_i_* is the spectral sensitivity function of receptor *i*, *I_S_* and *I_B_* are the spectral reflectance functions of a flower part (peripheral or central) and a green foliage background, respectively, and *D*_65_ (Wyszecki & Stiles, 1982) is the irradiance spectrum of CIE standard. The spectral sensitivity function *S_i_* was determined using the reported sensitivity function of photoreceptor classes in the retina of the swallowtail butterfly *P. xuthus* (Koshitaka et al., 2008, Supporting Information Figure S1a) or the Stavenga–Smits–Hoenders rhodopsin template (Stavenga, Smits, & Hoenders, 1993) with the sensitivity maxima (350 nm, 440 nm, and 525 nm) of crepuscular hawkmoth *D. elpenor* (Höglund et al., 1973; Schwemer & Paulsen, 1973, Supporting Information Figure S1b). The denominator in the calculation of *P_i_* standardizes the numerator by a green foliage background, considering that receptor sensitivity depends on a predominant background (i.e., green foliage) (Chittka & Kevan, 2005). This standardization by a green background is known to be less variable under the change of illumination spectrum from daytime to night (Johnsen et al., 2006). Thus, we only used the single illumination spectrum.

For butterflies with tetrachromatic vision (Koshitaka et al., 2008), flower color can be mapped into a three‐dimensional butterfly color space (Ohashi, Makino, & Arikawa, 2015). For this mapping, *P_i_* was transformed into the degree of receptor excitation *E_i_* describing the physiological input to the brain varying from 0 to 1 and being 0.5 for the green foliage background (Chittka, 1992):

$$E_{i}=\;\frac{P_{i}}{P_{i}+1}.$$

Then, *x*, *y*, and *z* coordinates in a three‐dimensional butterfly color space were calculated as follows (Ohashi et al., 2015):

$$x=\;E_{\text{uv}}-\;\frac{E_{\text{B}}+E_{\text{G}}+E_{\text{R}}}{3},$$

$$y=\;E_{\text{B}}-\;\frac{E_{\text{UV}}+E_{\text{G}}+E_{\text{R}}}{3},$$

$$z=\;E_{\text{G}}-\;\frac{E_{\text{UV}}+E_{\text{B}}+E_{\text{R}}}{3}.$$

For hawkmoths with trichromatic vision (Höglund et al., 1973; Schwemer & Paulsen, 1973), flower color is mapped on a Maxwell color triangle, which is a projection of the three‐dimensional color space with a plane of equal intensity (Kelber, Balkenius, & Warrant, 2003). The color coordinates *q_i_* in the Maxwell color triangle were calculated as.

$$q_{i}=\frac{P_{i}}{P_{\text{UV}}+P_{\text{B}}+P_{\text{G}}}.$$

To quantify the flower color difference contributing to pollinators’ preference, we applied a linear discriminant analysis to the flower colors of *H. fulva *and *H. citrina *in pollinators’ color space. First, we constructed the linear discriminant functions that discriminate *H. fulva* and *H. citrina* based on the color coordinates in a three‐dimensional color space of butterflies or hawkmoths for each of the central and the peripheral part. Second, to calculate the discriminant score, the color coordinates of all experimental flowers were applied to the discriminant functions. The discriminant scores of the central and the peripheral parts were used as quantitative variables of flower color: lower and higher discriminant scores represent *H. fulva*‐like and *H. citrina*‐like flower colors, respectively. The linear discriminant analysis was performed using the lda function of the MASS package (Venables & Riplay, 2002) under the R statistical environment version 3.4.4 (R Core Team, 2018).

To quantify the difference of color contrast contributing to pollinators’ preference, we used the Euclidean distance between coordinates in the color spaces that can be viewed as the chromatic distance (Balkenius & Kelber, 2004; Chittka, 1992; Ohashi et al., 2015). We used the Euclidean distance between two color coordinates of the central and peripheral parts of an individual flower as a quantitative variable of color contrast of the bullseye pattern (“bullseye contrast”), and the Euclidean distance between color coordinates of the peripheral part of an individual flower and that of the green foliage background as a quantitative variable of the color contrast between a peripheral part of a tepal and the background (“background contrast”). Here, we define “flower colour” and “contrast” operationally as a variable measured by the above variables and test its correlation empirically.

#### 2.3.2 Flower color in human vision, scent intensity, and floral morphology

To compare with our previous studies (Hirota et al., 2012, 2013), the flower color in human vision was also assessed by recording the flower color of the central part (not the peripheral part) by a simple matching with the standard color chart (SCC) of the Royal Horticultural Society, London, England. Scent intensity was measured immediately after flower opening by a handheld odour meter (OMX‐SR; Shinyei, Japan) (Hirota et al., 2012, 2013). The measurements were performed for at least three flowers per plant and then averaged. Two morphological traits significantly affecting pollination success (e.g., pollinator preference, Hirota et al., 2013) were measured before observations commenced: corolla orientation (the angle between a flower's main axis (from the floral base to the tip of pistil) and the horizontal) and stem height (from the ground to the top of an inflorescence).

### 2.4 Design of experimental arrays and pollinator observations

In the foraging experiments, an experimental array was composed of 36 potted plants of *Hemerocallis* randomly arranged in a 6 × 6 square with a distance of 50 cm between each pot and placed inside a net cage or outside in the experimental field of the Department of Biology, Kyushu University where swallowtail butterflies (*Papilio *spp.) and crepuscular hawkmoths (*Theretra *spp.) were observed in previous studies (Hirota et al., 2012, 2013). Additionally, nocturnal hawkmoth, *Agrius convolvuli*, was common (Hirota personal observation). We randomly selected one flower and cut off all remaining ones just before the observation if a plant had two or more flowers. We replaced some of the 36 plants with new ones day by day because the longevity of a flower is only half a day, and each individual plant did not flower everyday. The following Experiments 1, 2, and 3 were performed from 20 July‐2 August 2010, from 11–29 July 2014, and from 18–29 July 2015, respectively. These dates were around the flowering peak of the two *Hemerocallis* species.

#### 2.4.1 Experiment 1: innate preference of swallowtail butterflies

In Experiment 1, we examined the innate preference of swallowtail butterflies for floral traits. A naive individual of *Papilio xuthus *was released to the experimental array in each observation because *P. xuhus* is one of the major pollinators of *H. fulva* in that field (Hirota et al., 2012, 2013). Second‐ and third‐instar larvae of *P. xuthus* were collected at Fuchu Campus, Tokyo University of Agriculture and Technology (Tokyo, Japan). The collected larvae were reared on fresh citrus leaves at 25°C under a 16‐hr:8‐hr light:dark regime. Pupae were allowed to emerge at 25°C in a plastic box under a 16‐hr:8‐hr light:dark regime.

An experimental array was placed in a 7.0 × 4.5 m net cage with a height of 2.0 m. To provide trait variations large enough to detect significant preferences for floral traits, we designed the experimental array consisting of 18 potted plants of *H. fulva* and F2 hybrids that opened flowers in the morning of the observation day. Because we could not continue to provide 36 pots of diurnally flowering F2 hybrids throughout the experimental period, we used *H. fulva* in half of the experimental flowers. The trait values of experimental plants are shown in Supporting Information Table S1. The naive butterflies on the next day of emergence were used in the experiment after keeping them away from feeding. Only one butterfly was released at a time and allowed to fly freely in the net cage and then caught after 5 min of the latest visitation. One observer watched an experimental array and recorded pollinator visitation sequence. Simultaneously, we recorded pollinator behavior with High‐Definition Video Camera Recorder (XL H1; Canon, Japan). The observation was performed from 10:00 to 18:34 that corresponded to the flower‐visiting time of swallowtail butterflies (Hirota et al., 2012).

#### 2.4.2 Experiment 2: preference of crepuscular and nocturnal hawkmoths in the field

In Experiment 2, we examined the preference of crepuscular and nocturnal hawkmoths for floral traits in the field. An experimental array placed in the experimental field consisted of 36 potted plants of F2 hybrids that opened flowers in the evening on the day and were not visited by any insects at the start of observations. The trait values of experimental plants are shown in Supporting Information Table S1. Foraging behaviors of pollinators were recorded by an infrared video camera recorder (DVS A10FHDIR, Kenko, Japan) with two LED infrared illuminators (850 nm, IRSK02‐BK, Fuloon, China). The infrared video records enabled us to observe hawkmoth behavior through illumination but we could not identify species due to low resolution and monochrome images of the video records. We performed video observations each day from 18:30 until 24:00. The sunset time at Fukuoka for the experimental period was 19:21 – 19:32.

#### 2.4.3 Experiment 3: preference of swallowtail butterflies and crepuscular hawkmoths in the field

In our previous studies, hawkmoths showed a condition‐dependent preference for flower color using a *H. fulva*‐biased experimental array: Their preferences for flower color are possibly influenced by diurnal pollinators through the distribution of nectar source (Hirota et al., 2012, 2013). In Experiment 2, the influence of diurnal pollinators was excluded by using unvisited flowers. In Experiment 3, we examined whether stronger bullseye contrast characteristic of *H. citrina* is preferred by crepuscular hawkmoths under the existence of diurnal butterflies. To compare with the previous study, we used the composition of the experimental array of Hirota et al. (2012, 2013) that mimicked the situations in which mutants having *H. citrina*‐like floral traits appeared in a lower frequency within a diurnal population like *H. fulva*. An experimental array placed in the experimental field consisted of 24 and 12 potted plants of *H. fulva* and F2 hybrids, respectively, that opened in the morning on the observation day. The trait values of experimental plants are shown in Supporting Information Table S1. Foraging behaviors of diurnal pollinators were recorded by High‐Definition Video Camera Recorder, and those of crepuscular pollinators were recorded by an infrared video camera recorder with two LED infrared illuminators. We performed video observations each day from 9:00 to 20:30 because the start‐to‐close time of *H. fulva* varied from 18:00 to 20:30 with a peak at 20:30 (Nitta et al., 2010).

### 2.5 Data analysis and statistics

We defined a trip of pollinator foraging as a process from the arrival of one pollinator at the experimental array to its departure from the array. Bayesian generalized linear mixed‐effects models, including random effects for Trip ID, were used to analyse the effects of floral traits on the pollinator visitation rates. The response variable was the visitation rate of hawkmoths or butterflies per flower per foraging trip. The explanatory variables were flower colors (the discriminant scores of both central and peripheral parts), bullseye contrast, scent intensity, corolla orientation, and stem height. Flower colors and flower contrasts were calculated based on hawkmoth or butterfly color vision. If some explanatory variables correlated strongly, those variables except one were excluded from the analysis. We assumed a Poisson error structure and a log link. In Experiment 2, the analyses were performed using two types of the dataset. The dataset 1 including the whole foraging trips of all hawkmoths was used to examine the average preference of hawkmoths. The start‐to‐close time of *H. fulva* peak at 20:30 and the start‐to‐open time of *H. citrina* peak at 18:30 (Nitta et al., 2010). Until early night (until around 20:30), the flowers of both *H. fulva* and *H. citrina* are open, and after that, the flowers of *H. citrina* is only open. Thus, the dataset was divided into two time periods: from 18:30 until 20:30 (dataset 2c) and from 20:30 until 24:00 (dataset 2n).

If the 95% Bayesian credible interval (CI) for a partial regression coefficient included zero, the corresponding explanatory variable had a non‐significant effect and was classified into the no effect group. If the CI did not include zero, the corresponding explanatory variable had a significant effect and was classified into the negative or the positive effect group depending on the sign of the median of the posterior distribution of each regression coefficient. For better convergence in parameter estimation, all explanatory variables were standardized to mean = 0 and *SD* = 1.

All models were fitted in the R statistical environment version 3.4.4 (R Core Team, 2018), using Stan version 2.17.3 (Stan Development Team, 2018), a Hamiltonian Monte Carlo sampler. Results are based on 12,000 samples each from three chains, after 2,000 burn‐in steps in each chain. The convergence of the Markov chains was checked with $\hat{R}$ (Gelman, Carlin, Stern, & Rubin, 2004) for each parameter. The $\hat{R}$ values obtained were less than 1.002 for all parameters. The mean and 95% Bayesian credible interval for each parameter were evaluated using the MCMC samples. Model code was generated using rstanarm (Stan Development Team, 2016), a package for Rstan. The data were analyzed using uninformative priors.

In four of six analyses, two or more explanatory variables were significant. To visualize the influence of a particular explanatory variable on a response variable while adjusting for the influence of the other traits, we did not plot observed response variables but predicted values, based on the observed value of the explanatory variable of interest and the mean values of the other explanatory variables, to which we added the residuals from the model (Worley & Harder, 1996). Consequently, the data points in the figures cannot be directly comparable among figures. While Worley and Harder (1996) made adjustments using normal mixed models, we here adopted the adjustment using Poisson mixed‐effect models (detailed in Supporting Information Methods).

## 3 RESULTS

### 3.1 Difference of flower color and contrast between *H. fulva* and *H. citrina*

**Figure 3:**
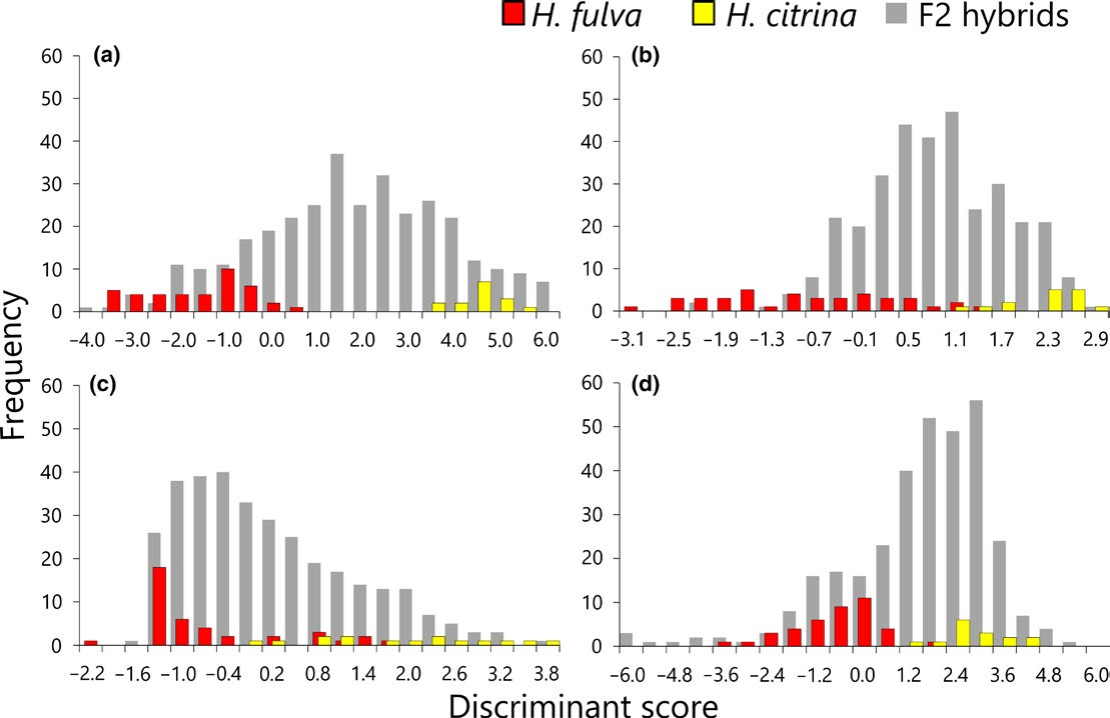
Histogram of the discriminant scores of the central (a, c) and the peripheral parts (b, d) based on color visions of swallowtail butterflies (a, b) and hawkmoths (c, d). Red, yellow, and gray bars represent the distribution of *H. fulva*, *H. citrina*, and F2 hybrids, respectively

We quantified flower color variation using the discriminant scores. The bimodal pattern of the discriminant scores depended on the pollinator color visions and differed between the central and peripheral parts. In the butterfly color vision, the discriminant scores of the central part were bimodally distributed and there was no overlap between *H. fulva* (from −3.898 to 0.130) and *H. citrina* (3.292 to 5.221, Figure 3a). The discriminant scores of the peripheral part of the two species slightly overlapped (*H. fulva*: −3.426 to 1.194, *H. citrina*: 1.076 to 2.737, Figure 3b). In the hawkmoth color vision, the discriminant scores of the two species showed larger overlap in both the central and peripheral parts (the central part: *H. fulva*: −2.379 to 1.463, *H. citrina*: −0.298 to 3.419; the peripheral part: *H. fulva*: −3.758 to 1.583, *H. citrina*: 1.0268 to 3.806), and the overlap in the central part was larger than in the peripheral part (Figure 3c,d).

**Figure 4:**
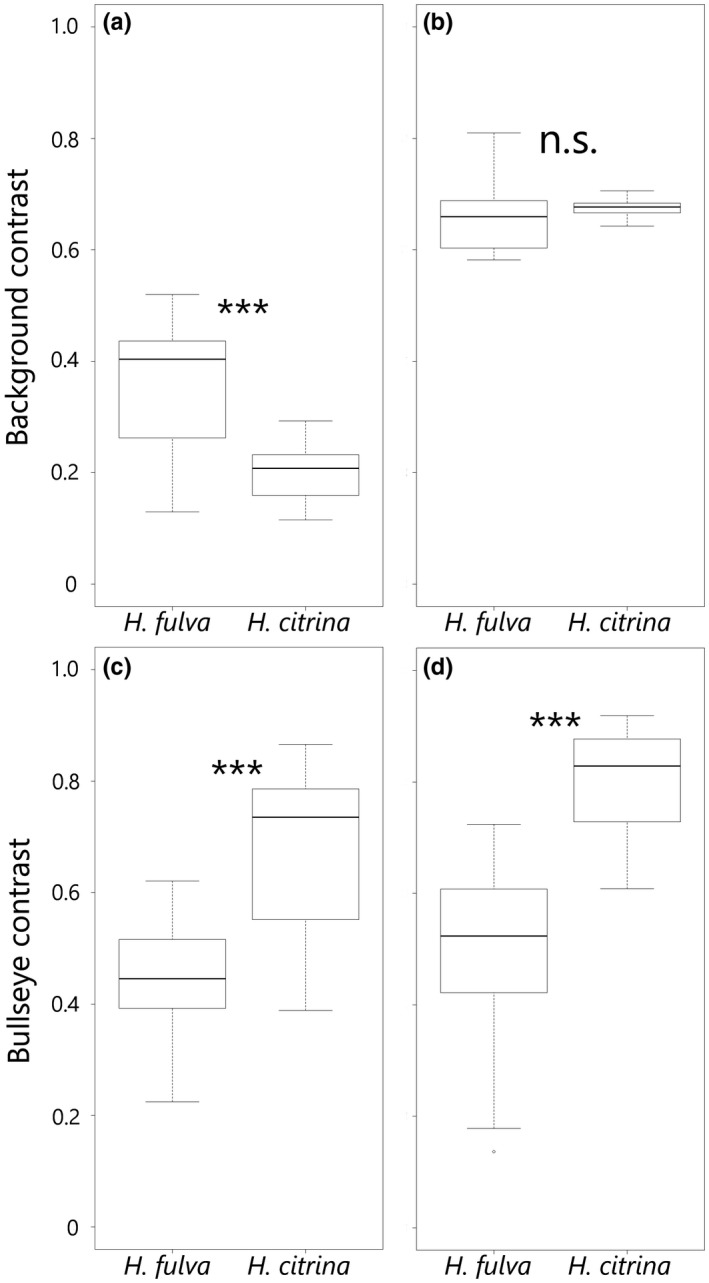
Box plots of the color contrasts of *H. fulva* and *H. citrina*. The upper panel shows the color contrasts between the peripheral part of a tepal and the green foliage background (background contrast) based on swallowtail butterfly vision (a) and hawkmoth vision (b). The lower panel shows the color contrasts between the peripheral and central parts of a tepal (bullseye contrast) based on swallowtail butterfly vision (c) and hawkmoth vision (d). Indicated are median, quartiles, whiskers. The differences between *H. fulva* and *H. citrina* were examined by two‐sided *t* tests. Three asterisks indicate the difference is significant (*p* < 0.001)

For the background contrast in the butterfly vision, *H. fulva* showed significantly larger values than *H. citrina* (*H. fulva*: mean ± *SE* = 0.359 ± 0.018, *H. citrina*: 0.195 ± 0.020, *t* test, *t* = 4.265, *df* = 47, *p* < 0.001, Figure 4a). For the background contrast in the hawkmoth color vision, there was no significant difference between *H. fulva* and *H. citrina* (*H. fulva*: 0.661 ± 0.009, *H. citrina*: 0.677 ± 0.007, *t* test, *t* = −0.836, *df* = 47, *p* = 0.408, Figure 4b). For the bullseye contrast in the butterfly color vision, *H. citrina* showed significantly larger values than *H. fulva* (*H. fulva*: 0.443 ± 0.016, *H. citrina*: 0.677 ± 0.052, *t* test, *t* = −5.542, *df* = 47, *p* < 0.001, Figure 4c). Also for the bullseye contrast in the hawkmoth color vision, *H. citrina* showed significantly larger values than *H. fulva* (*H. fulva*: 0.505 ± 0.023, *H. citrina*: 0.800 ± 0.033, *t* test, *t* = −5.712, *df* = 47, *p* < 0.001, Figure 4d).

### 3.2 Correlations among floral traits

In F2 hybrids, the correlations among flower color and color contrasts varied with the models of pollinator color visions (Supporting Information Figures S2 and S3). In the butterfly color vision, the discriminant score of the peripheral part was strongly correlated with the background contrast (Pearson's product moment correlation coefficient *r* = −0.867, *t* = −30.87, *p* < 0.001, Supporting Information Figure S2). The discriminant score of the central part was significantly correlated with that of the peripheral part (*r* = 0.539, *t* = 11.31, *p* < 0.001) and the background contrast (*r* = −0.498, *t* = −10.15, *p* < 0.001). The other combinations of traits, including the scent intensity and morphological traits, were only weakly correlated. In the hawkmoth color vision, the discriminant score of the peripheral part was strongly correlated with the bullseye contrast (*r* = 0.767, *t* = 21.14, *p* < 0.001, Supporting Information Figure S3). The correlations of the other combinations were not significant. The color for human vision (SCC) was correlated with both of the discriminant scores in the butterfly color vision but not or weakly correlated with the two discriminant scores in the hawkmoth color vision.

Among the explanatory variables, the discriminant score of the peripheral part was strongly correlated with the bullseye contrast in hawkmoth color vision. Thus, the discriminant score of the peripheral part was excluded from the statistical models of hawkmoth's preference.

### 3.3 Experiment 1: innate preference of swallowtail butterflies

**Figure 5:**
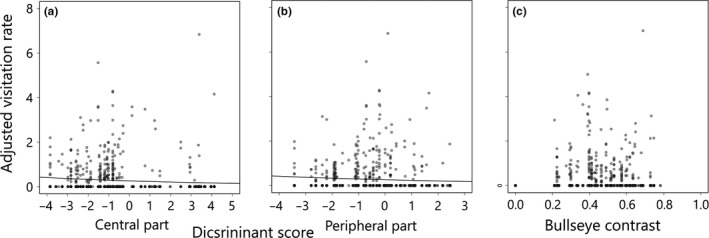
Effect of flower colors of central (a) and peripheral parts (b) and bullseye contrast (c) on visitation rate of naive swallowtail butterflies in Experiment 1. The flower colors of the central (a) and peripheral part (b) were defined by discriminant analysis: large and small values of the discriminant score represent *H. citrina*‐ and *H. fulva*‐like flower colors. Data points of each experimental flower are shown by transparent gray and the overlaps of points by darker color. A solid line indicates the prediction of the model if the explanatory variable was significant. All points and a solid line are adjusted by controlling for the effects of the other explanatory variables (detailed in Supporting Information Methods)

Twenty‐five individuals of naive *P. xuthus* foraged the experimental array and visited cumulatively 358 experiment flowers, including 281 flowers of *H. fulva* and 77 flowers of F2 hybrids. The visitation rate significantly decreased with the discriminant scores of both the central and peripheral parts (Table 1; the lower CI was larger than zero), indicating that *H. fulva*‐like flower color was preferred (Figure 5a,b). The visitation rate also significantly increased with stem height but decreased with the scent intensity. Contrastingly, the visitation rate was not significantly affected by the bullseye contrast (Figure 5c).

**Table 1:** Medians, standard deviations, and 95% credible intervals of the posterior distribution of the partial regression coefficients in the Bayesian generalized linear mixed effects model for innate butterfly preference (Experiment 1)

Explanatory variables	Coefficients	*SD*	2.5%	97.5%
Discriminant score of central part	**−0.247**	0.096	−0.435	−0.058
Discriminant score of peripheral part	**−0.170**	0.079	−0.323	−0.015
Bullseye contrast	−0.009	0.071	−0.146	0.132
Scent intensity	**−0.547**	0.119	−0.791	−0.325
Corolla orientation	0.104	0.061	−0.015	0.224
Stem height	**0.386**	0.061	0.267	0.504

Bold values indicate that the parameters have significant positive or negative effects on the visitation rate.

### 3.4 Experiment 2: preference of crepuscular and nocturnal hawkmoths in the field

**Figure 6:**
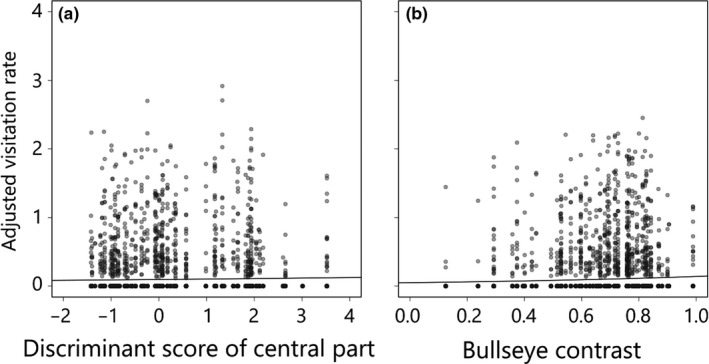
Effect of flower color of central part (a) and bullseye contrast (b) on visitation rate of hawkmoths using the whole foraging trips of all hawkmoths in Experiment 2. The flower colors of the central part (a) were defined by discriminant analysis: large and small values of the discriminant score represent *H. citrina*‐ and *H. fulva*‐like flower colors. The flower color of the peripheral part was excluded from the statistical analysis considering its strong correlation with the bullseye contrast under the hawkmoth color vision. Data points of each experimental flower are shown by transparent gray and the overlaps of points by darker color. A solid line indicates the prediction of the model if the explanatory variable was significant. All points and solid lines are adjusted by controlling for the effects of the other explanatory variables (detailed in Supporting Information Methods)

During 15 days of the experiment, we observed 180 foraging trips and 944 cumulative visitations of hawkmoths for which species were not identified. The analysis of the whole observation period (dataset 1) showed that the visitation rate of hawkmoths significantly increased with the discriminant score of the central part and the bullseye contrast (Table 2; the upper CI was lower than zero), indicating that *H. citrina*‐like colored flowers with stronger bullseye pattern were preferred. Figure 6b is consistent with those statistical results although Figure 6a does not show a clear increase in the visitation rate with the discriminant score. In contrast, hawkmoth visitation rate was not significantly affected by scent intensity.

**Table 2:** Medians, standard deviations, and 95% credible intervals of the posterior distribution of the partial regression coefficients in the Bayesian generalized linear mixed effects model for crepuscular and nocturnal hawkmoth preference (Experiment 2)

Explanatory variables	Coefficients	*SD*	2.5%	97.5%
Discriminant score of central part	**0.075**	0.035	0.006	0.145
Bullseye contrast	**0.188**	0.042	0.105	0.270
Scent intensity	0.015	0.033	−0.050	0.079
Corolla orientation	−0.039	0.036	−0.109	0.033
Stem height	**−0.086**	0.036	−0.156	−0.015

Bold values indicate that the parameters have significant positive or negative effects on the visitation rate.

Observed hawkmoths included both crepuscular and nocturnal species; 107 trips (566 visitations) and 73 trips (378 visitations) of hawkmoths were observed before and after 20:30, respectively (Supporting Information Figure S4). The visitation rates of both crepuscular and nocturnal hawkmoths (dataset 2c, 2n) significantly increased with the bullseye contrast (Tables 3 and 4; the upper CI was lower than zero), indicating that stronger bullseye contrast was preferred (Supporting Information Figure S5b,d). Although the visitation rate did not clearly increase with the discriminant score of the central part (Supporting Information Figure S5a,c), the posterior distribution of the partial regression coefficient of the discriminant score was biased for positive in dataset 2c, 2n (but 95% CI included zero). This indicates that *H. citrina*‐like colored flower was marginally preferred by crepuscular and nocturnal hawkmoths. Additionally, the visitation rate of nocturnal hawkmoths significantly decreased with corolla orientation.

**Table 3:** Medians, standard deviations, and 95% credible intervals of the posterior distribution of the partial regression coefficients in the Bayesian generalized linear mixed effects model for crepuscular hawkmoth preference (dataset 2c from Experiment 2)

Explanatory variables	Coefficients	*SD*	2.5%	97.5%
Discriminant score of central part	0.064	0.045	−0.025	0.153
Bullseye contrast	**0.184**	0.054	0.079	0.291
Scent intensity	0.045	0.045	−0.043	0.132
Corolla orientation	0.020	0.046	−0.070	0.109
Stem height	−0.057	0.045	−0.145	0.03

Based on the start‐to‐close time of *H. fulva* flower, we defined the hawkmoths that foraged before 20:30 as crepuscular hawkmoths. Bold values indicate that the parameters have significant positive or negative effects on the visitation rate.

**Table 4:** Medians, standard deviations, and 95% credible intervals of the posterior distribution of the partial regression coefficients in the Bayesian generalized linear mixed effects model for nocturnal hawkmoth preference (dataset 2n from Experiment 2)

Explanatory variables	Coefficients	*SD*	2.5%	97.5%
Discriminant score of central part	0.095	0.056	−0.019	0.202
Bullseye contrast	**0.193**	0.067	0.065	0.329
Scent intensity	−0.023	0.052	−0.128	0.077
Corolla orientation	**−0.132**	0.059	−0.248	−0.018
Stem height	**−0.133**	0.059	−0.249	−0.018

Based on the start‐to‐close time of *H. fulva* flower, we defined the hawkmoths that foraged after 20:30 as nocturnal hawkmoths. Bold values indicate that the parameters have significant positive or negative effects on the visitation rate.

### 3.5 Experiment 3: preference of swallowtail butterfly and crepuscular hawkmoth in the field

During 9 days of the experiment, we observed 16 foraging trips of swallowtail butterflies (*P. xuthus*, *P. helenus,* and *P. memnon*) and 25 trips of hawkmoths. Swallowtail butterflies visited 24 cumulative flowers (21 flowers of *H. fulva* and three flowers of F2 hybrids) and hawkmoths visited 77 cumulative flowers (49 flowers of *H. fulva* and 28 flowers of F2 hybrids).

The visitation rates of butterflies significantly decreased with scent intensity (Table 5). The 95% CI of the partial regression coefficients of the other traits included zero, indicating that swallowtail butterflies did not show any significant preference for these traits including flower colors (discriminant scores) (Supporting Information Figure S6). The hawkmoth visitation rates significantly increased with bullseye contrast (Supporting Information Figure S7b) and decreased with scent intensity (Table 6). However, there was no obvious decrease in visitation rate with flower color (discriminant score) in Supporting Information Figure S7a.

**Table 5:** Medians, standard deviations, and 95% credible intervals of the posterior distribution of the partial regression coefficients in the Bayesian generalized linear mixed effects model for wild swallowtail butterfly preference (Experiment 3)

Explanatory variables	Coefficients	*SD*	2.5%	97.5%
Discriminant score of central part	−0.317	−0.328	−1.174	0.427
Discriminant score of peripheral part	−0.189	−0.184	−0.758	0.427
Bullseye contrast	0.155	0.158	−0.419	0.754
Scent intensity	**−1.533**	−1.606	−3.309	−0.285
Corolla orientation	−0.176	−0.163	−0.673	0.402
Stem height	0.299	0.301	−0.301	0.914

Bold values indicate that the parameters have significant positive or negative effects on the visitation rate.

**Table 6:** Medians, standard deviations, and 95% credible intervals of the posterior distribution of the partial regression coefficients in the Bayesian generalized linear mixed effects model for crepuscular hawkmoth preference (Experiment 3)

Explanatory variables	Coefficients	*SD*	2.5%	97.5%
Discriminant score of central part	**−0.272**	0.143	−0.572	−0.010
Bullseye contrast	**0.412**	0.129	0.164	0.672
Scent intensity	**−0.361**	0.154	−0.676	−0.078
Corolla orientation	−0.163	0.114	−0.378	0.069
Stem height	0.120	0.125	−0.124	0.367

Bold values indicate that the parameters have significant positive or negative effects on the visitation rate.

## 4 DISCUSSION

In butterfly and hawkmoth vision, both *H. fulva* and *H. citrina* had a bullseye pattern and the bullseye contrast of *H. citrina* was significantly stronger than that of *H. fulva* (Figures 2 and 3c,d). Both crepuscular and nocturnal hawkmoths showed a significant preference for higher bullseye contrast whereas the hawkmoths showed weak or no preference for *H. citrina*‐like central color (Tables 2, 3, 4, 6). This finding supports our hypothesis that hawkmoths use a higher bullseye contrast as a foraging cue more consistently than the other floral traits. In contrast, the preferences of swallowtail butterflies were affected not by the bullseye contrast but by the central and peripheral flower color (Table 1).

This is the first demonstration that hawkmoths are attracted by the floral bullseye pattern in the field. Hawkmoths showed consistent preference to stronger bullseye pattern independent from the composition of the experimental array and the influence of diurnal pollinators. Thus, we suggest that individuals with intensified bullseye contrast were advantageous to be pollinated by hawkmoths during the process of evolution from *H. fulva*‐like ancestor to *H. citrina*. In laboratories, it has been known that hawkmoths can recognize floral patterns. First, *Macroglossum stellatarum* preferred artificial flowers with the ring (not striped) pattern to those with uniform pattern (Kelber, 2002). Second, hawkmoths probed on the yellow‐colored and brighter area of a striped or crossed pattern (Goyret, 2010; Goyret & Kelber, 2012). However, the presence and function of the floral bullseye pattern in natural nocturnal flowers have not been demonstrated probably because our understanding about the visual system of nocturnal pollinators had been limited and we human lack UV perception. Recently, it has been reported that some nocturnal animals have highly sensitive eyes (reviewed in Warrant, 2008; Kelber & Lind, 2010) and can discriminate color under dim light conditions (Kelber, Balkenius, & Warrant, 2002; Roth & Kelber, 2004; Somanathan, Borges, Warrant, & Kelber, 2008). Thus, we need to clarify how widely color vision is used in nocturnal animals and those studies will deepen our understanding of the evolution of nocturnal flowers.

In the hawkmoth color vision, the bullseye contrast was strongly correlated with the discriminant score of the peripheral part, but not with the discriminant score of the central part. This result implies that the intensity of the bullseye contrast of *Hemerocallis* is largely determined by the peripheral part having the UV reflectance. Although many flowers pollinated by nocturnal hawkmoths lack UV reflectance uniformly (Raguso et al., 2003; White et al., 1994), some yellow flowers pollinated by nocturnal hawkmoths have a UV bullseye pattern composed of UV‐absorbing and UV‐reflecting parts (Hirota et al., 2012; Kawano et al., 1995). This UV bullseye pattern may be disadvantageous in white flowers because UV‐absorbing white flowers show more reliable higher contrast against the background of green leaves under the various nocturnal conditions than the other colors (Johnsen et al., 2006). On the other hand, the UV bullseye pattern composed of UV‐absorbing and UV‐reflecting parts may be advantageous in yellow‐flowered species to attract hawkmoths by compensating the relatively less reliable lower contrast of yellow color to the green background. Additionally, the stronger bullseye contrast with intense UV reflectance is possibly advantageous at night. Koski and Ashman (2015) showed the deleterious effect of UV irradiance on pollen grain viability in UV bullseye flowers in the daytime, for the peripheral parts of curving petals can directly reflect UV to anthers. At night, UV irradiance is much weaker than in daytime so that nocturnal flowers could not suffer from reduced pollen viability by floral UV reflection relative to diurnal flowers.

The preferences of hawkmoths for flower color were incoherent between Experiments 2 and 3: hawkmoths marginally preferred *H. citrina*‐like central flower color in Experiment 2 (Table 2) but they preferred *H. fulva*‐like central flower color in Experiment 3 (Table 6). In Experiment 2, the absolute value of the partial regression coefficient of the discriminant score was lower than the coefficient of the bullseye contrast, indicating that flower color has a smaller effect on hawkmoth attraction than bullseye contrast. Under our experimental settings, the nectar availability at crepuscule was influenced by the abundance of the diurnal pollinators and the composition of the experimental array. In Experiment 3, *H. fulva*‐like colored flowers were dominant in the experimental array and the visitation rate of butterflies to the array was low. This situation corresponds to Hirota et al. (2013) that showed that hawkmoths preferred human reddish flowers over yellowish flowers using a *H. fulva*‐biased experimental array consisting of unvisited flowers. We suggest that this low visitation rate resulted because *H. fulva*‐like colored flowers kept nectar until crepuscule and hawkmoths learned the association between *H. fulva*‐like color and nectar avalability. It has been documented that hawkmoths can be trained to switch their color preference by learning an association of a certain color with a nectar reward (Balkenius & Kelber, 2006; Goyret, Pfaff, Raguso, & Kelber, 2008; Kelber et al., 2003). In the field, hawkmoth preferences for flower color, which are easily learned, should be largely influenced by the abundance of competitive pollinators, and the distribution of the remaining nectar source. More careful studies are needed to assess the magnitudes of selective pressures on attractive traits by considering the community level interaction and its annual fluctuation.

Available evidence supports that swallowtail butterflies can recognize the bullseye contrasts in two *Hemerocallis* species. First, using artificial flowers, Kandori and Ohsaki (1998) demonstrated that the bullseye pattern enhanced foraging efficiency and flower constancy of butterflies, *Pieris rapae*. Second, the wavelength discrimination ability of *Papilio* is the highest among the animals tested so far (Koshitaka et al., 2008). The bullseye contrast intensities of *H. fulva* and *H. citrina* were 0.443 ± 0.016 and 0.677 ± 0.052, respectively. This is significantly larger than 0.03, a criterion for perceptible color discrimination in butterfly vision (Ohashi et al., 2015). Thus, the presence of perceptible bullseye pattern of *Hemerocallis* is expected to be used by butterflies. However, its intensity did not show any significant effect on butterfly attraction. This discrepancy can be explained by assuming that swallowtail butterflies have a threshold of response and non‐response to the bullseye pattern, depending on the contrast intensity.

Naive swallowtail butterflies preferred *H. fulva*‐like flower color of both central and peripheral parts. This result supports our previous studies showing wild butterflies’ preference for reddish flower to human vision because both of the discriminant scores were negatively correlated with SCC (Supporting Information Figure S2). In Experiment 3, although most butterflies visited flowers of *H. fulva*, we did not detect the preference of wild butterflies for flower color. It may be caused by the small visitation rate and the *H. fulva*‐biased experimental array. The background contrast is stronger in *H. fulva* than in *H. citrina* (Figure 3a) and negatively correlated with the discriminant score of the peripheral part (Supporting Information Figure S3). *Papilio xuthus* uses the target‐background intensity contrast when landing (Koshitaka, Arikawa, & Kinoshita, 2011). Thus, swallowtail butterflies could land more easily on *H. fulva*‐like colored peripheral flower parts than on *H. citrina‐*like colored peripheral parts. The swallowtail butterflies preferred not only peripheral color but also central color. The center part of *H. fulva* flower reflects longer wavelength light than the center part of *H. citrina* (Figure 2). Most insects, such as bees and hawkmoths, lack a red receptor (Lunau & Maier, 1995), but swallowtail butterflies have a red receptor and can perceive longer wavelength (Kinoshita et al., 1999). It is more costly for at least bees to feed on the flowers that reflect only longer wavelength, such as human red, than on other flowers with more conspicuous colors for them, like pink or yellow, in terms of searching time for flowers (Spaethe, Tautz, & Chittka, 2001) although long wavelength light (up to 650 nm) can stimulate a “green” (540 nm) receptor for the majority of bees only if the light is very strong (Chittka & Waser, 1997). This is probably the reason why many bees tend not to feed on red flowers (Rodríguez‐Gironés & Santamaría, 2004). In contrast, for butterflies, it is a better strategy to forage on flowers that reflect only longer wavelength which are seldom visited by bees.

Our study has uncovered the different effect of bullseye contrast on the attraction of diurnal and nocturnal pollinators by controlling the effect of background flower color. We revealed striking differences in the responses to flower color and the bullseye contrast between swallowtail butterflies and hawkmoths. This result indicates that the difference of visual systems between pollinators may have promoted floral divergence. There is increasing physiological evidence that pollinators use not only visual cue but also a variety of sensory information to find, feed on, and learn about flowers (e.g., von Arx, Goyret, Davidowitz, & Raguso, 2012; Clarke, Whitney, Sutton, & Robert, 2013). Further field observations based on knowledge about the varieties and differences of pollinator sensory systems will provide profitable clues to understand floral evolution mediated by pollinators.

## AUTHORS CONTRIBUTIONS

SKH, NM, AAY, and TY: conceived and designed the study. SKH and NM: collected the data and analyzed it. SKH: wrote the manuscript, with the contribution of all authors.

## DATA ACCESSIBILITY

Data with the floral traits of the experimental flowers and pollinator visitations are openly available in Dryad (https://doi.org/10.5061/dryad.7b3c73k).
