# Supplementary material for: UV bullseye contrast of Hemerocallis flowers attracts hawkmoths but not swallowtail butterflies
Source: Ecol Evol. 2018 Dec 18;9(1):52–64. doi: 10.1002/ece3.4604 (PMC6342183; doi:10.1002/ece3.4604)
Supplement: Supplementary file 1 [file ECE3-9-52-s001.docx]

**Supporting Information**

**Supplementary methods, tables and figures**

**UV bullseye contrast of *Hemerocallis* flowers attracts hawkmoths but not swallowtail butterflies.**

Shun K. Hirota, Nozomu Miki, Akiko A. Yasumoto and Tetsukazu Yahara

* Corresponding author: shun.hirota.c7@tohoku.ac.jp

**Contents**

**Supporting Methods** Adjustment of response variables in the figures

**Table S1** Traits of experimental populations.

**Fig. S1** Relative sensitivities of the photoreceptors of *Papilio xuthus* and *Deilephila elpenor*.

**Fig. S2** Correlations among floral traits in butterfly colour vision in F2 hybrids

**Fig. S3** Correlations among floral traits in hawkmoth colour vision in F2 hybrids.

**Fig. S4** Visitation rate of hawkmoths in each time zone in experiment 2.

**Fig. S5** Effect of flower colour of central part and bullseye contrast on visitation rate of crepuscular and nocturnal hawkmoths in experiment 2.

**Fig. S6** Effect of flower colours of central and peripheral parts and bullseye contrast on visitation rate of wild swallowtail butterflies in experiment 3.

**Fig. S7** Effect of flower colour of central part and bullseye contrast on visitation rate of crepuscular hawkmoths in experiment 3.

**Supporting Methods** Adjustment of response variables in the figures

To visualize the influence of a particular explanatory variable on a response variable by controlling for the effects of the other explanatory variables, previous studies (e.g., Worley and Harder 1996) adopted an adjustment by adding the residuals to the values predicted from general linear models.

$$Y_{adjusted}=Y_{predicted}+Residuals =\alpha+\beta_{f}x_{f}+\sum\beta_{i}\bar{x}_{i}+Y_{observe}-\left( \alpha+\beta_{f}x_{f}+\sum{\beta_{i}x}_{i} \right)=Y_{observed}+\sum\left( \beta_{i}\bar{x}_{i}-{\beta_{i}x}_{i} \right),$$

where $Y_{adjusted}$ and $Y_{observed}$ are the adjusted and observed visitation rates, respectively, $\alpha$ is the intercept, $\beta_{f}$ is the partial regression coefficient of the focal explanatory variable, $\beta_{i}$ is the partial regression coefficient of the nonfocal explanatory variable *i*, $x_{f}$ and $x_{i}$ are the standardized observed values of focal and nonfocal explanatory variables, respectively, and $\bar{x}_{i}$ is the average value of the nonfocal explanatory variable *i*.

In this study, we used a generalized linear model with a Poisson error structure and a log link. Under a log link function, the effects of the explanatory variables are multiplicative rather than additive. $\bar{x}_{i}$ is equal to zero because of standardization. Thus, adjusted values are given by the following calculation:

$$\log\left( Y_{adjusted} \right)=\alpha+\beta_{f}x_{f}+\sum\beta_{i}\bar{x}_{i}+\log\left( Y_{observed} \right)-\left( \alpha+\beta_{f}x_{f}+\sum{\beta_{i}x}_{i} \right)$$

$$=\log\left( Y_{observed} \right)+\sum\left( \beta_{i}\bar{x}_{i}-{\beta_{i}x}_{i} \right)$$

$$=\log\left( Y_{observed} \right)-\sum\beta_{i}x_{i}$$

, $Y_{adjusted}=Y_{observed}e^{-\sum\beta_{i}x_{i}}$.

This adjustment was made only with pollinator-visited flowers. Because of multiplicative effects of the explanatory variables, the visitation rates of unvisited flowers are represented by zero in the figures.

**References**

Worley, A. C., and L. D. Harder. 1996. Size-dependent resource allocation and costs of reproduction in *Pinguicula vulgaris* (Lentibulariaceae). *Journal of Ecology,* **84**, 195-206. doi: 10.2307/2261355

**Table S1** Trait values of experimental populations. In experiment 1, the population consisted of 18 potted plants each of *H. fulva* and F2 hybrids. In experiment 2, the population consisted of 36 potted plants of F2 hybrids. In experiment 3, the population consisted of total 24 and 12 potted plants of *H. fulva* and F2 hybrids, respectively.

| Traits | Experiment 1 | | | | Experiment 2 | | | | Experiment 3 | | | |
| --- | --- | --- | --- | --- | --- | --- | --- | --- | --- | --- | --- | --- |
|  | mean | SD | min. | max. | mean | SD | min. | max. | mean | SD | min. | max. |
| Flower colour for human vision (SCC) | 17.52 | 6.99 | 2 | 23 | 10.47 | 4.91 | 3 | 23 | 19.55 | 5.47 | 3 | 25 |
| Discriminant score of central part based on |  |  |  |  |  |  |  |  |  |  |  |  |
| Swallowtail butterfly vision | -0.526 | 2.172 | -3.846 | 4.119 | - | - | - | - | -1.148 | 1.994 | -3.898 | 3.719 |
| Hawkmoth vision | - | - | - | - | 0.249 | 1.159 | -1.422 | 3.523 | -0.766 | 0.918 | -2.379 | 2.692 |
| Discriminant score of peripheral part based on |  |  |  |  |  |  |  |  |  |  |  |  |
| Swallowtail butterfly vision | -0.404 | 1.443 | -3.426 | 2.456 | - | - | - | - | -0.434 | 1.169 | -3.426 | 2.354 |
| Hawkmoth vision | - | - | - | - | 1.286 | 2.003 | -6.157 | 4.522 | -0.403 | 1.751 | -3.758 | 4.713 |
| Bullseye contrast based on |  |  |  |  |  |  |  |  |  |  |  |  |
| Swallowtail butterfly vision | 0.464 | 0.158 | 0.013 | 0.782 | - | - | - | - | 0.470 | 0.144 | 0.110 | 0.755 |
| Hawkmoth vision | - | - | - | - | 0.65 | 0.181 | 0.074 | 0.989 | 0.539 | 0.174 | 0.135 | 0.913 |
| Scent intensity | 8.40 | 8.89 | 0.1 | 42.1 | 11.54 | 6.99 | 0.3 | 39.8 | 6.00 | 7.09 | 0.1 | 33.7 |
| Corolla orientation (degree) | 56.4 | 13.8 | 0 | 89 | 57.4 | 13.4 | 10 | 89 | 43.6 | 25.9 | -70 | 90 |
| Stem height (cm) | 99.7 | 15.4 | 48 | 147 | 107.8 | 15.3 | 70 | 149 | 93.5 | 18 | 47 | 141 |


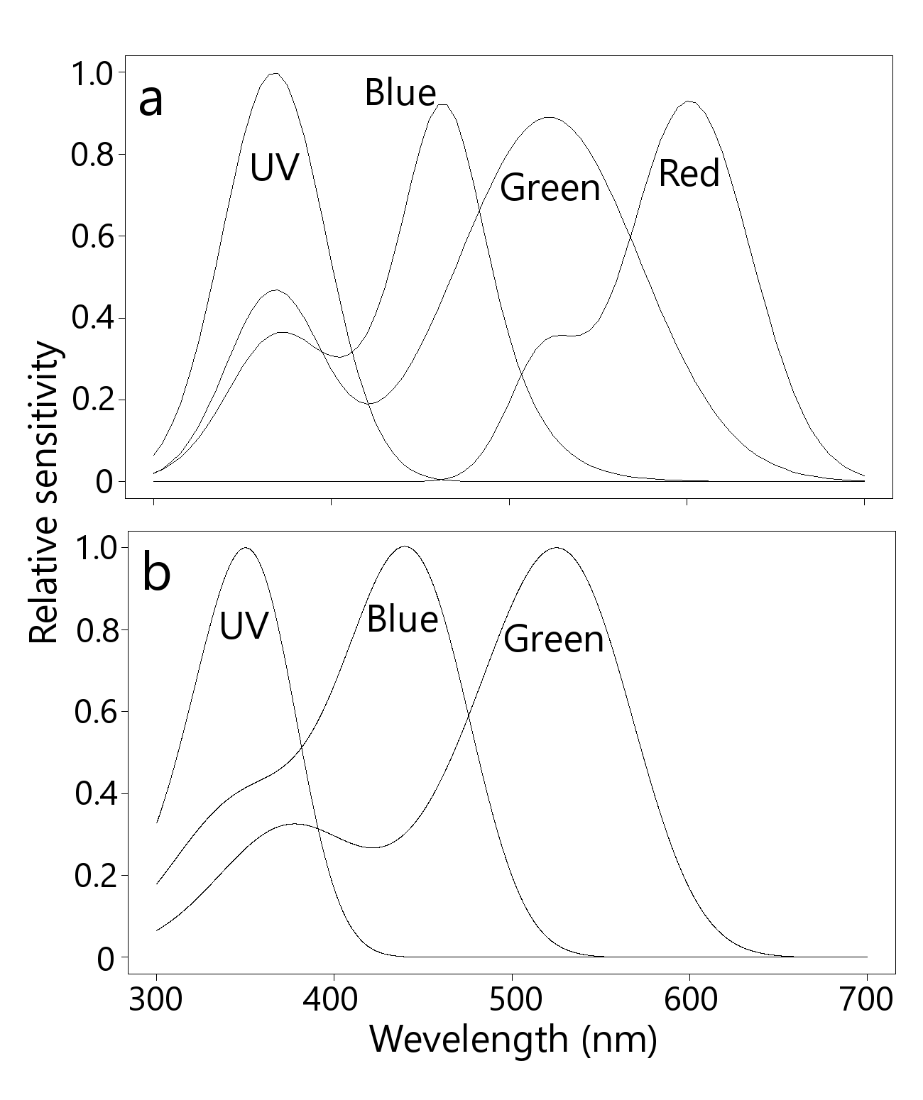


**Fig. S1** Relative sensitivities of the photoreceptors of *Papilio xuthus* (a) and *Deilephila elpenor* (b). In panel a, UV, Blue, Green and Red refer to the photoreceptors with peak absorption wavelengths of 365, 465, 515, and 600 nm, respectively. In panel b, UV, Blue and Green refer to the photoreceptors with peak absorption wavelengths of 350, 440, and 525 nm, respectively.

**
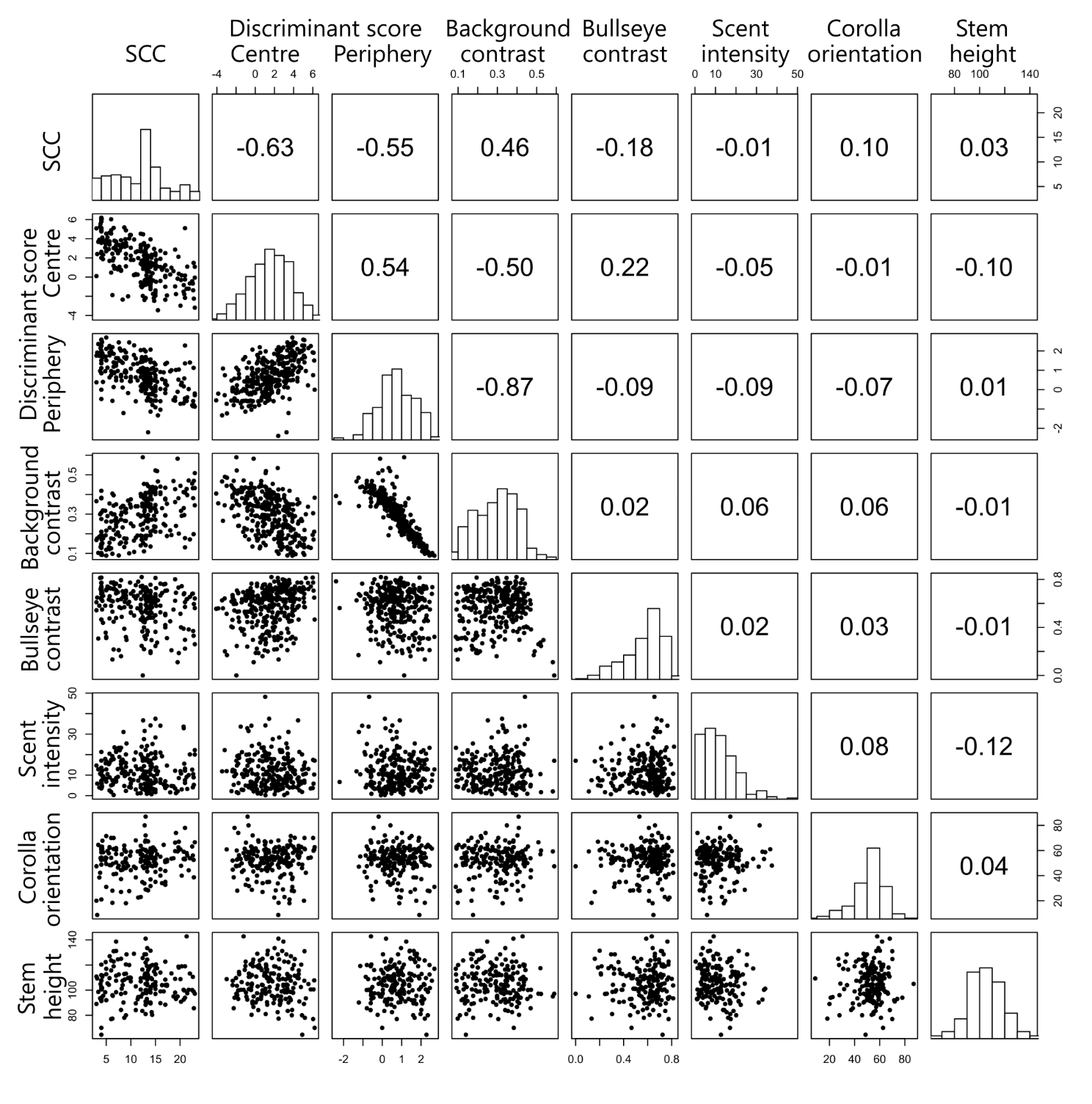
**

**Fig. S2** Correlations among floral traits in butterfly colour vision in F2 hybrids. The panels show a scatter plot of matrices (SPLOM), with bivariate scatter plots below the diagonal, histograms on the diagonal, and the Pearson correlation coefficients above the diagonal. Each dot represents the average trait values of a genet. The discriminant scores and the contrasts were calculated based on butterfly colour vision.

**
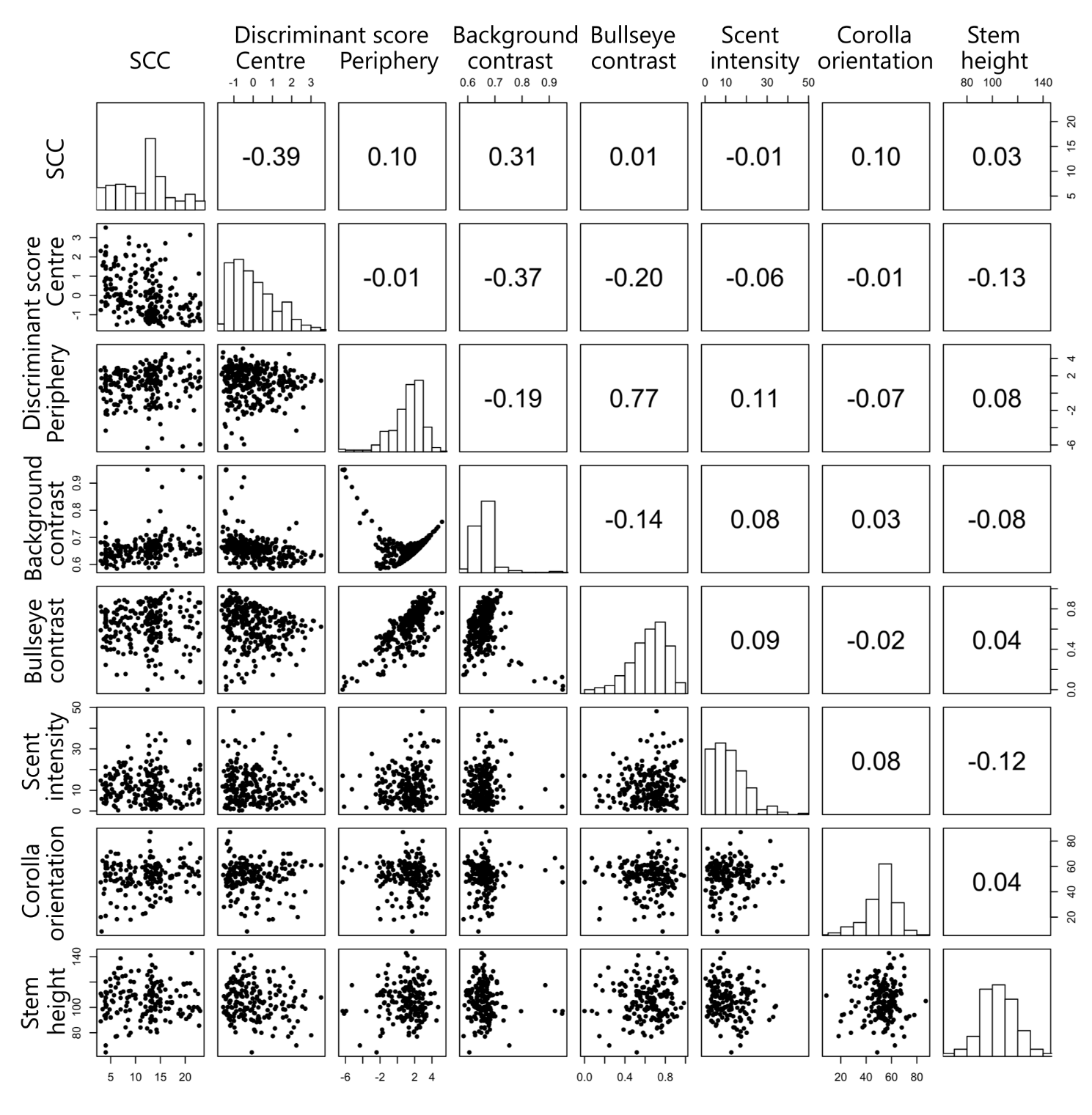
**

**Fig. S3** Correlations among floral traits in hawkmoth colour vision in F2 hybrids. The panels show a scatter plot of matrices (SPLOM), with bivariate scatter plots below the diagonal, histograms on the diagonal, and the Pearson correlation coefficients above the diagonal. Each dot represents the average trait value of a genet. The discriminant scores and the contrasts were calculated based on hawkmoth colour vision.


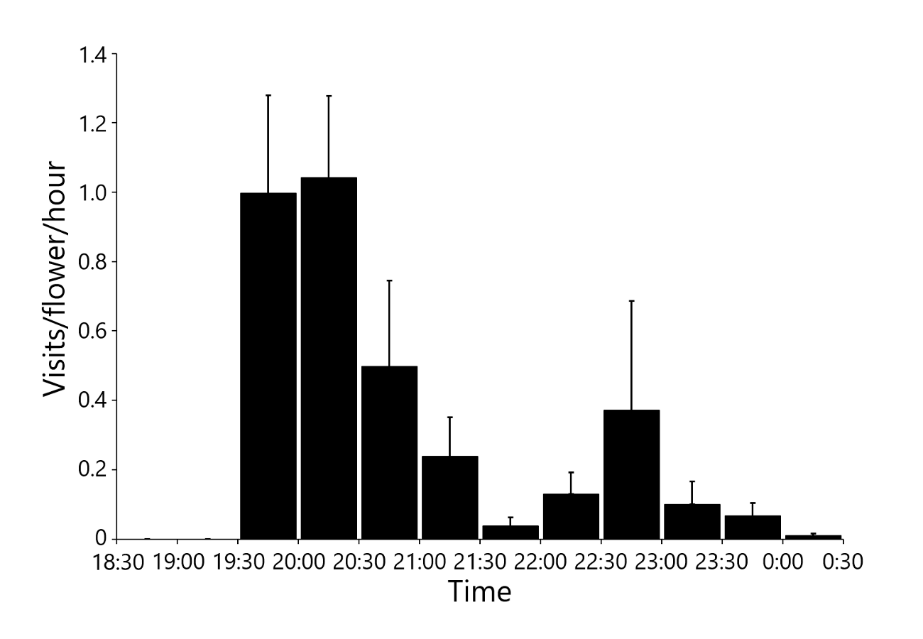


**Fig. S4** Visitation rate of hawkmoths in each time zone in experiment 2.


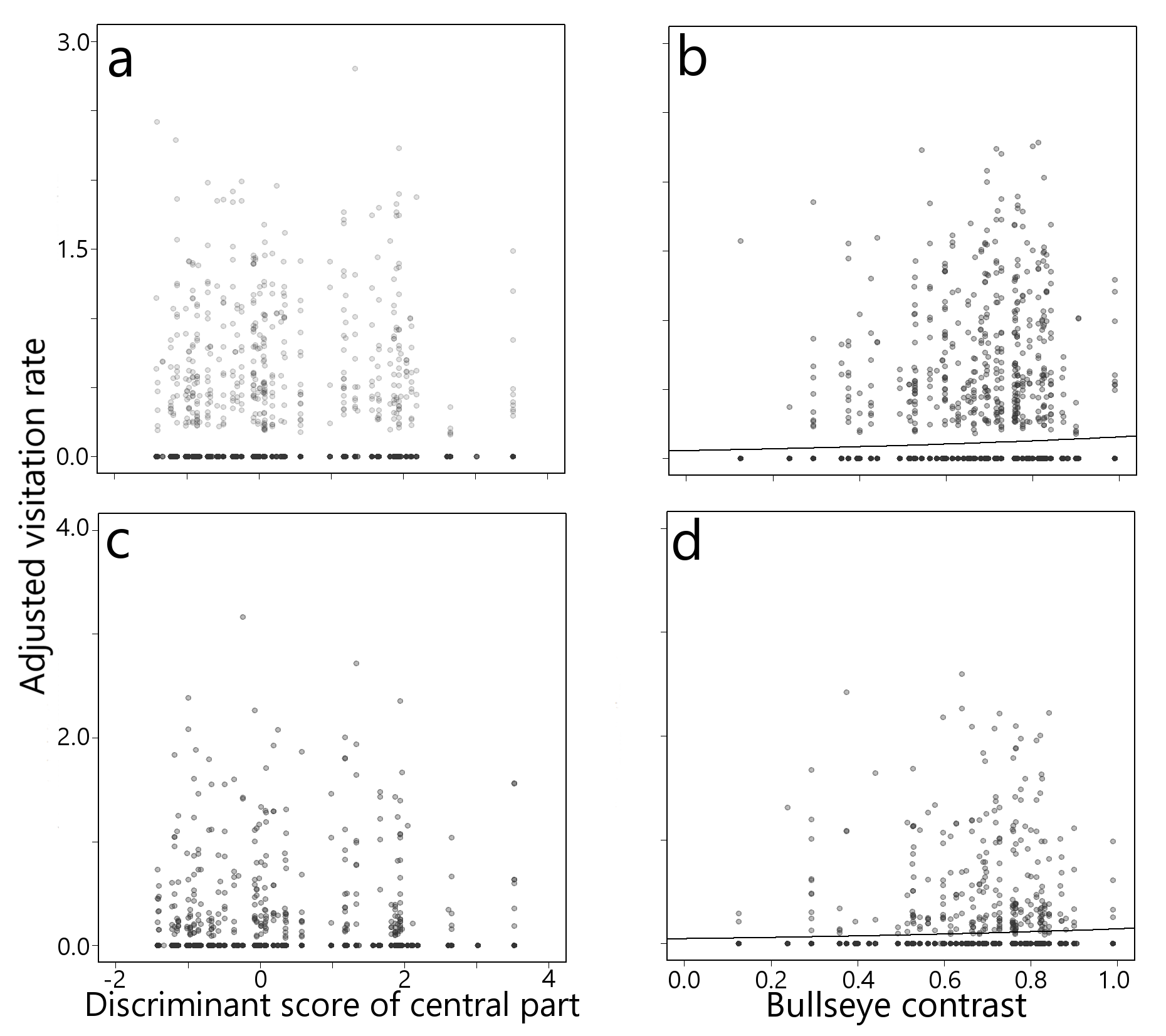


**Fig. S5** Effect of flower colour of central part (a, c) and bullseye contrast (b, d) on visitation rate of crepuscular (upper panel) and nocturnal (lower panel) hawkmoths in experiment 2. In experiment 2, the data set was divided into two time periods: from 18:30 until 20:30 (crepuscular hawkmoths) and from 20:30 until 24:00 (nocturnal hawkmoths) based on flowering time of *H. fulva* and *H. citrina.* Data points of each experimental flower are shown by transparent gray and the overlaps of points by darker colour. A solid line indicates the prediction of the model if the explanatory variable was significant. All points and solid lines are adjusted by controlling for the effects of the other explanatory variables.


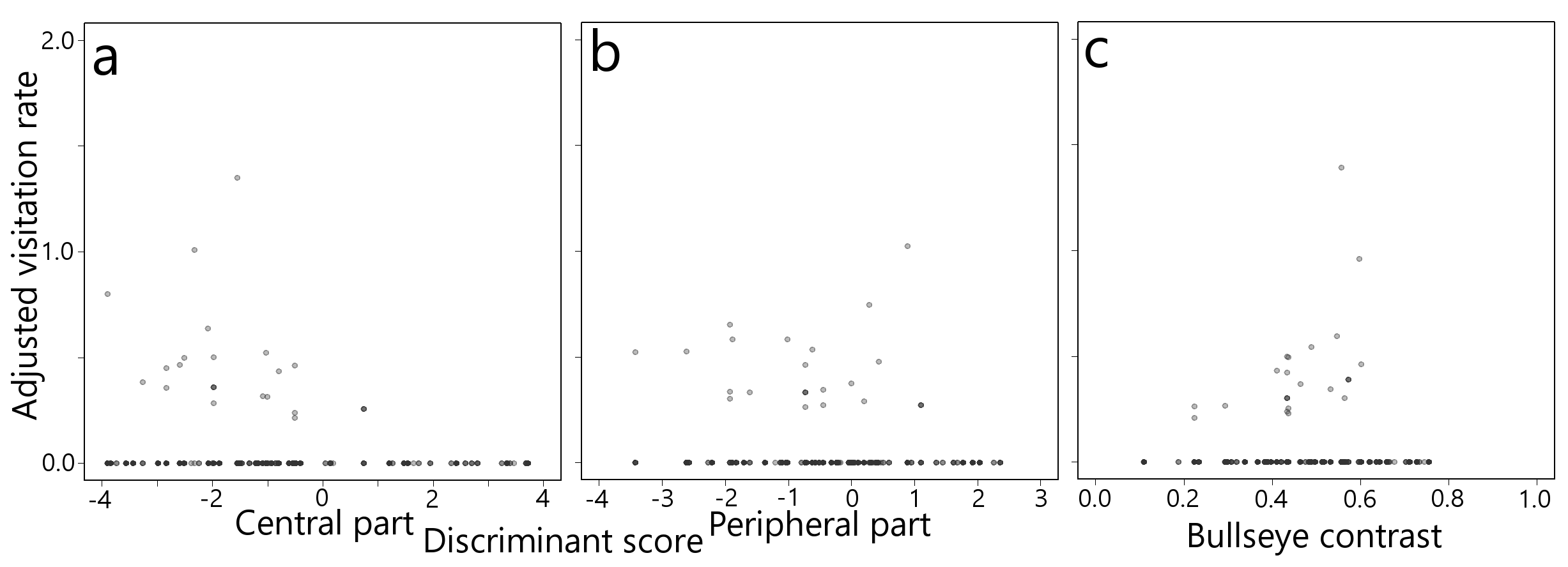


**Fig. S6** Effect of flower colours of central (a) and peripheral parts (b) and bullseye contrast (c) on visitation rate of wild swallowtail butterflies in experiment 3. Data points of each experimental flower are shown by transparent gray and the overlaps of points by darker colour. A solid line indicates the prediction of the model if the explanatory variable was significant. All points and solid lines are adjusted by controlling for the effects of the other explanatory variables.


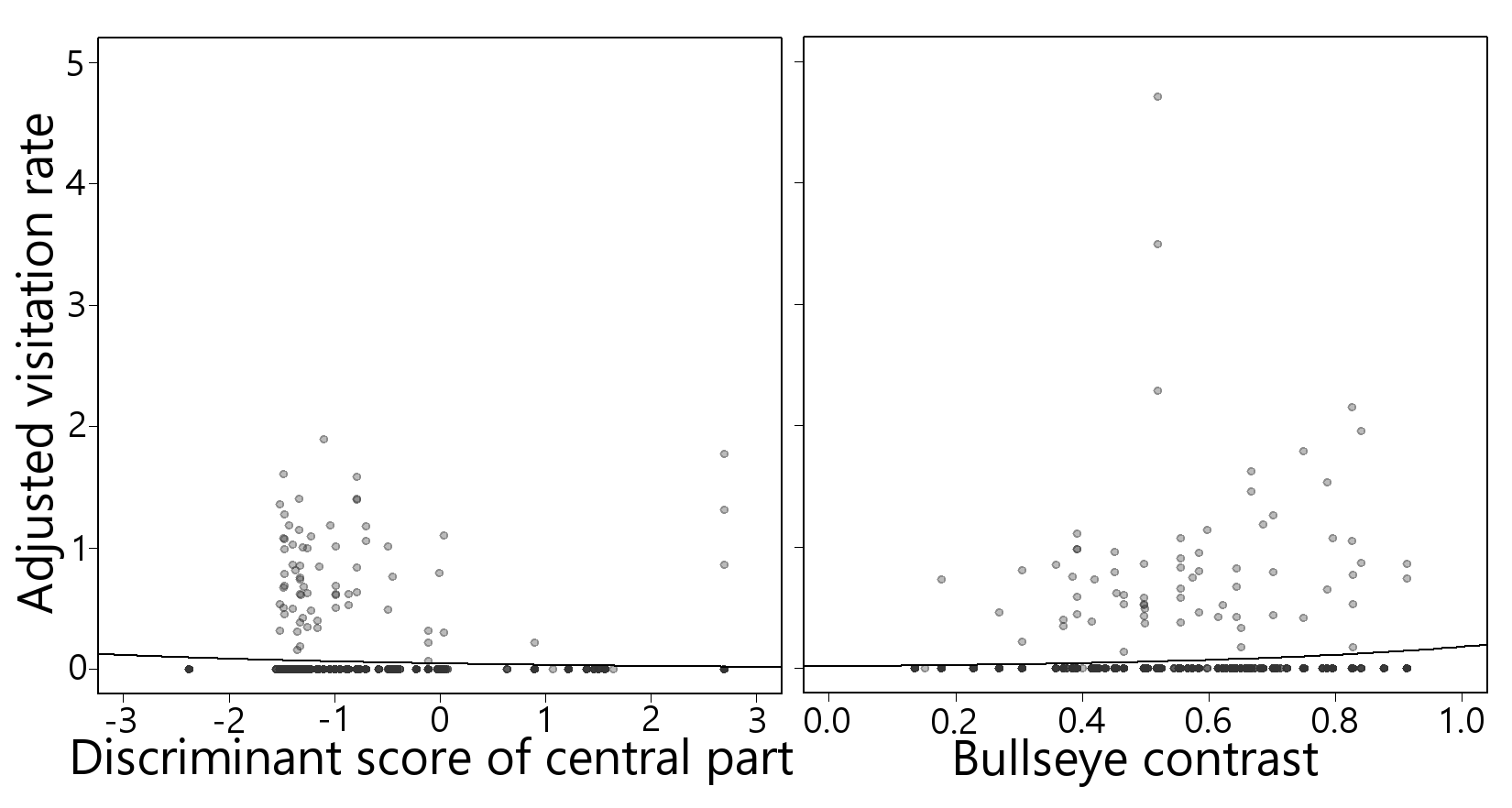


**Fig. S7** Effect of flower colour of central part (a) and bullseye contrast (b) on visitation rate of crepuscular hawkmoths in experiment 3. Data points of each experimental flower are shown by transparent gray and the overlaps of points by darker colour. A solid line indicates the prediction of the model if the explanatory variable was significant. All points and solid lines are adjusted by controlling for the effects of the other explanatory variables.
